# T Cell Specificity: A Great Challenge in Chagas Disease

**DOI:** 10.3389/fimmu.2021.674078

**Published:** 2021-06-29

**Authors:** Fátima Ferragut, Gonzalo R. Acevedo, Karina A. Gómez

**Affiliations:** Laboratorio de Inmunología de las Infecciones por Tripanosomátidos, Instituto de Investigaciones en Ingeniería Genética y Biología Molecular (INGEBI), Consejo Nacional de Investigaciones Científicas y Técnicas (CONICET), Buenos Aires, Argentina

**Keywords:** Chagas disease, *Trypanosoma cruzi*, T cell specificity, mouse model, human model

## Abstract

The CD4^+^ and CD8^+^ T cell immune response against *T. cruzi*, the parasite causing Chagas disease, are relevant for both parasite control and disease pathogenesis. Several studies have been focused on their phenotype and functionally, but only a few have drilled down to identify the parasite proteins that are processed and presented to these cells, especially to CD4^+^ T lymphocytes. Although approximately 10,000 proteins are encoded per haploid *T. cruzi* genome, fewer than 200 T cell epitopes from 49 *T. cruzi* proteins have been identified so far. In this context, a detailed knowledge of the specific targets of T cell memory response emerges as a prime tool for the conceptualization and development of prophylactic or therapeutic vaccines, an approach with great potential to prevent and treat this chronic disease. Here, we review the available information about this topic in a comprehensive manner and discuss the future challenges in the field.

## Introduction

Chagas disease, caused by the infection with the protozoan *Trypanosoma cruzi*, is a neglected tropical disease from the American continent that has spread from the limits established by vector ecology due to human migration (1) to non-endemic places such Canada, USA, Europe, Australia and Japan (2). Last estimates calculate that about 6-8 million people are infected in the world (1), with more than 70 million people living in areas at risk for infection (3).

**Figure 1:**
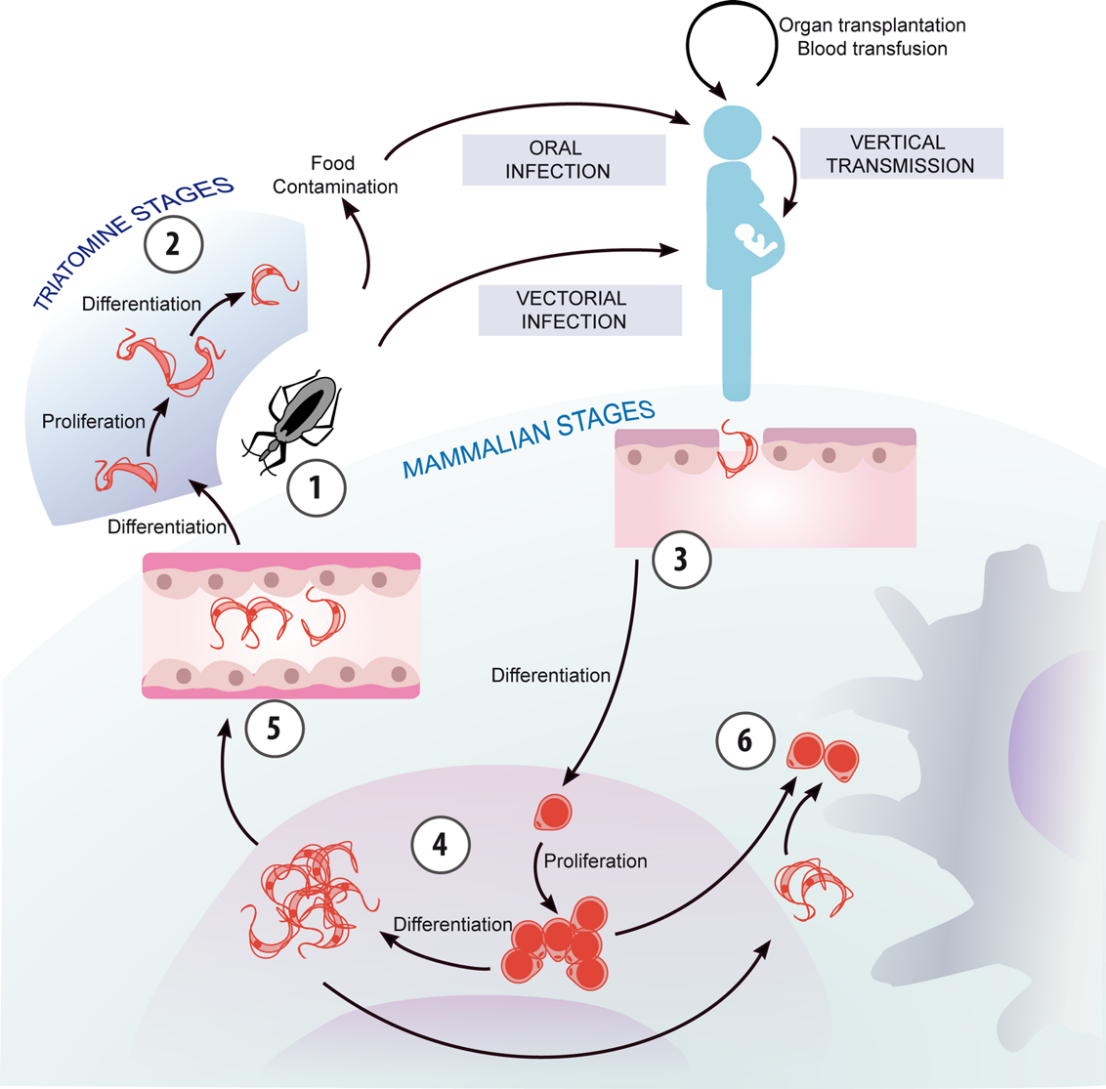
Chagas disease transmission and parasite life cycle. The life cycle of *T. cruzi* can be conceptualized with its beginning at moment when the triatomine vector ingests bloodstream trypomastigotes from an infected mammalian host, which differentiate into replicative epimastigotes (1). Epimastigotes migrate to the hindgut and differentiate into non-replicative, infectious metacyclic trypomastigotes (2), which are excreted with the feces of the vector and are ready to infect a new mammalian host. Additionally, *T. cruzi* can be transmitted through other routes than vectorial spread, such as mother-to-child and oral transmission besides blood transfusion and organ transplantation. Trypomastigotes invade host cells and differentiate into the intracellular, replicative amastigote form (3), which can also transform into trypomastigotes (4). These are released when the host cells break down, and may invade new cells, migrate through the bloodstream, or be ingested by a vector (5). In addition, extracellular amastigotes, originated by the premature rupture of the host cell or by extracellular trypomastigote differentiation, can be engulfed by macrophages (6).

The parasite can be transmitted through other routes than vectorial spread and its life cycle includes different intermediate hosts and developmental stages (**Figure 1**). When vectorially acquired, Chagas disease shows two major phases. The acute phase typically lasts for about 2 months and presents a high number of parasites circulating in the blood, but in most cases symptoms are absent, mild or unspecified (4) -for instance prolonged fever, headache, myalgia, lymphadenitis, hepatomegaly, and splenomegaly-. However, visible signs of infection such as skin lesion (chagoma) or a swollen eyelid (the so-called Romaña sign) can help in the diagnosis of new cases (1). If patients are not treated, a chronic phase follows, during which most people remain asymptomatic but infected for life. Nonetheless, it is estimated that up to 40% of chronically infected patients can develop organ involvement, being cardiomyopathy and megaviscera (megaoesophagus, megacolon, or both) the most prevailing (5). Based on these phases, some diagnostic tests are better suited than others. In the acute phase, the high parasitemia makes it possible to identify the presence of circulating parasites by the direct microscopic visualization of blood samples or by polymerase chain reaction (PCR)-based diagnostics methods. In contrast, serologic testing is preferred in the chronic phase when parasitemia is low. Since no single standard reference test is available, diagnosis should be based on the presence of IgG against various *T. cruzi* antigens by the use of at least two serological assays based on different principles, such as indirect fluorescent assay, indirect hemagglutination, and ELISA. And, a third assay is then indicated to clarify infection status when serology results are discordant and samples can yield persistent inconclusive results (5).

With regard to Chagas disease treatment, two drugs -benznidazole and nifurtimox- are currently available. Despite their effectiveness in the acute phase, there is no consensus for their use in the chronic phase. Furthermore, the drugs prolonged duration and the side effects, which include gastric intolerance, skin rashes or neuromuscular problems, can lead to treatment discontinuation and failure (6, 7). Thus, the recent clinical trial BENDITA (Benznidazole New Doses Improved Treatment & Therapeutic Associations) aimed to find effective regimes that produce fewer adverse effects and improve patients´ adherence to treatment (8). This work demonstrated the efficacy of the treatment protocol duration of 2 weeks with a daily dose of 300 mg/day of benznidazole after the 12-month follow-up (8). In summary, in chronic phase, generally a symptomatic supportive treatment is performed. Chagas disease patients with cardiomyopathy are treated with specific medication to control cardiac damage or with an implantable cardioverter defibrillator (9). Heart transplantation is the only course of action in case of advanced tissue failure, which is an expensive procedure, limited by the lack of donors and with a risk of reactivation of parasitaemia due to the use of immunosuppressive drugs. In patients with digestive involvement, conservative or even surgical treatment is indicated depending on the stage of the disease (2).

## Specific T Cell Response in Chagas Disease

**Figure 2:**
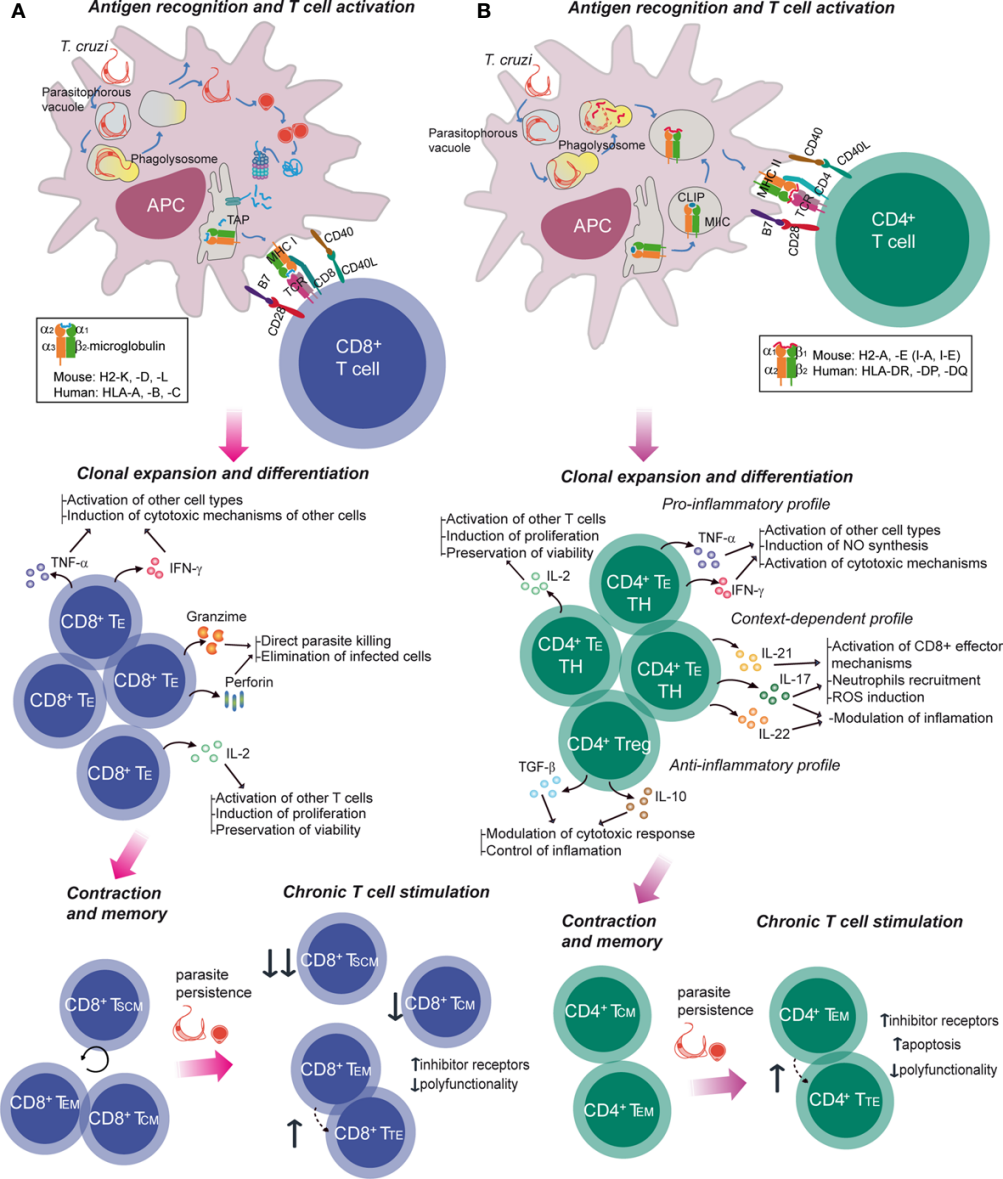
Specific T cell response in Chagas disease. The activation of T-cell immune responses is mediated by APCs, which deliver antigen-specific and costimulatory signals. *Trypanosoma cruzi* trypomastigote invades the host cell or is passively internalized, leading to the formation of the parasitophorous vacuole which fuses with a lysosome resulting in a phagolysosome. The parasite can escape to the cytoplasm **(A)** where it differentiates into the amastigote form and its proteins are targeted for proteosomal degradation. Peptides generated are then imported into the endoplasmic reticulum (ER) to be presented to CD8^+^ T cells in the context of MHC class I molecules. These molecules consists of a β2-microglobulin and a polymorphic alpha chain which contains the peptide binding groove and it is encoded by one of the mouse *H2-K*, *H2-D* and *H2-L* class I MHC genes and human *HLA-A*, *-B* or *–C* class I MHC genes. **(B)** Class II MHC is made up of polymorphic α and β chains that form the peptide binding groove, each encoding in mouse *H2-A* and *H2-E* class II MHC loci and in human *HLA-DR*, *-DP* and *-DQ* class II MHC loci. In the class II MHC pathway, proteins from parasite are degraded and antigenic peptides are loaded onto the binding cleft from the MHC. Peptide-MHC complexes are then presented in the host cell surface to the CD4^+^ T cell. In both CD8^+^ and CD4^+^ T cells, activation generates the expansion and differentiation into effector specific T cells (T_E_), their survival and function being sustained by cytokines and soluble mediators. Following antigen clearance, contraction takes place with a numerical reduction of antigen-specific cells. A subset of the activated T cells survives beyond this phase and differentiates into long-lasting memory T cells. During chronic infections, which entail persistent antigen exposure and/or inflammation, different degrees of dysfunctionality can be observed, including terminal differentiation and T cell exhaustion with progressive loss of effector functions and co-expression of inhibitor receptors. IL, interleukin; IFN, interferon; TNF, tumor necrosis factor; T_H_, T helper; Treg, regulatory T cell; T_SCM_, stem cell memory T cell.

Upon infection with the parasite, mammal hosts develop both innate and adaptive immune responses that play a major role during the acute and chronic phases of the disease (10). Regarding T cells, CD8^+^ cytotoxic T lymphocytes have been predominantly studied (11), given that the replicative phase of *T. cruzi* life cycle within mammals host takes place in the intracellular environment (**Figure 2**). The activation of these T cells results in the acquisition of the molecular machinery for the elimination of the target cell, which includes cytoplasmic granules containing proteins such as perforin and granzymes and cytokine production, like IFN-γ, TNF-α and IL-2. In chronic Chagas disease, several types of dysfunction have been described. Thus, concomitant with terminal differentiation, CD8^+^ T cells from patients with severe disease present a higher frequency of cells coexpressing inhibitory receptors, and a lower frequency of polyfunctional parasite-specific CD8^+^ T cells (11). Besides its role in the elimination of the parasite, some evidence suggests that CD8^+^ T cells are involved in tissue damage and inflammatory processes linked to the clinical manifestations of Chagas disease (10).

Much less is known about CD4^+^ T cells involvement (**Figure 2**), possibly because their ultimate action requires the collaboration with other cells (10). These cells are characterized by the expression of surface molecules and the secretion of cytokines that modulate the activity of other cells, mainly macrophages, DCs and other lymphocytes. The majority of proliferating activated CD4^+^ T cells differentiate into one of the multiple T_H_ profiles, acquiring a diversity of effector properties. It has been suggested that a coordinated response between T_H_1 and T_H_2 profiles is desirable, with predominance of T_H_1 effector mechanisms for the control/elimination of the parasite (10). In addition, the subset of regulatory T cells (T_reg_), which operate tolerance mechanisms, is increased in asymptomatic patients compared to those with cardiac or digestive symptoms. Furthermore, CD4^+^ T cells develop some signatures of T cell response exhaustion in the chronic phase of the disease (10).

The specific responses of both types of T cells depend on the processing and presentation of *T. cruzi* peptides by antigen presenting cells (APC) and their recognition by the T cell receptor (TCR). For this reason, studies on new therapeutic approaches (12–15) attempt to understand the mechanisms underlying the interaction between peptide-MHC (pMHC, MHC: major histocompatibility complex) complexes and TCR, and also functional and phenotypic characteristics of antigen-specific T cells, which can be investigated by diverse strategies (16–23). In Chagas disease, different *T. cruzi* targets that can be processed and presented to T cells have been evaluated. Extensive research is mainly focused on members of the *T. cruzi trans*-sialidase (TS) superfamily, the largest *T. cruzi* gene family. These proteins are distributed along the cell body, flagellum and flagellar pocket of the parasite. This superfamily includes not only active *trans*-sialidases that transfer sialic acid from host glycoconjugates to the parasite membrane proteins, but also several proteins with unknown function, among others. In addition, non-*trans*-sialidase (non-TS) T cell activating antigens are also analyzed.

Taking into consideration the central role that T cell response plays in Chagas disease and the potential use of antigen-specific T cell response data in disease prevention or treatment, this review revisits the results of published research on the specific response against *T. cruzi* epitopes recognized by CD8^+^ and CD4^+^ T cells. To this end, reports about a variety of *T. cruzi* antigens are discussed, with particular emphasis on peptides that trigger T cell response in mouse models or human samples. Additionally, we comment on what we consider the greatest challenges faced by scientists today in the study of T-cell specificity in the immune response against this parasite.

## Models for Chagas Disease Specific T Cell Response

**Figure 3:**
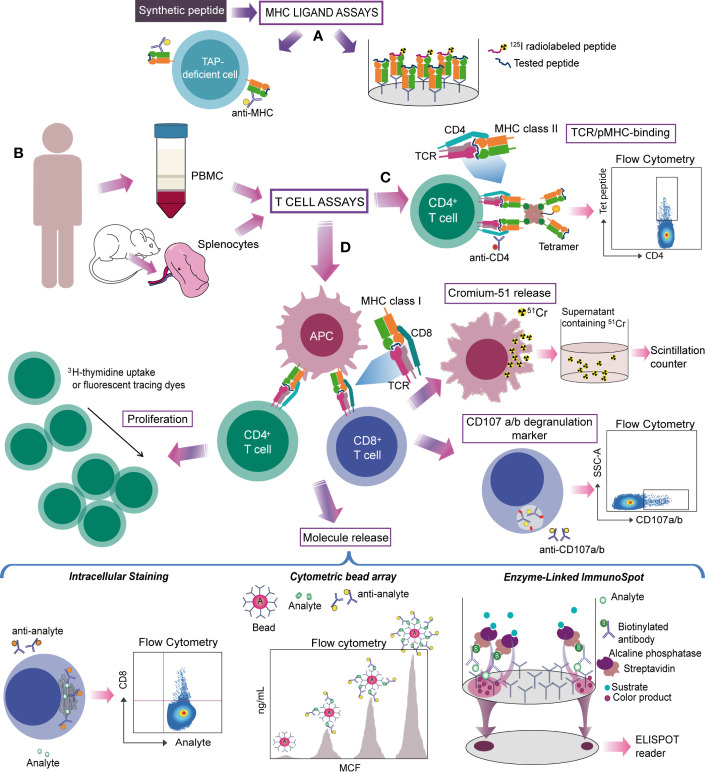
Strategies for studying antigen-specific T cells in Chagas disease. **(A)** Experimentally identified or predicted candidate target peptides are synthesized and tested by MHC ligand assays. MHC-peptide binding can be evaluated by TAP deficient cells (cells lacking the transporter associated with antigen-processing that fail to transport peptides into the endoplasmic reticulum, reducing MHC class I stability and surface expression) loaded with the peptide and stained for the MHC, or by the ability to inhibit the binding of a radiolabeled probe peptide. **(B)** Splenocytes from mouse, PBMC from human or CD4^+^- or CD8^+^-depleted PBMC are used to detect antigen-specific T cells trough T cell assays. **(C)** MHC-peptide multimer/tetramer is based on the ability of TCR to recognize the pMHC complex from CD8^+^ or CD4^+^ T cells. **(D)** Other strategies evaluate the specific T-cell response on the basis of a functional readout. Activated cytotoxic CD8^+^ T cells are commonly detected by measuring chromium in the supernatant released by killed target cells. Assays that determine secretion of cytolytic granule contents (granzyme or perforin) and/or labelling responding cells with antibodies to CD107a and b provide an alternative way to measure cytolytic activity. On CD4^+^ T cells activation, the amplification of antigen-specific populations is tested through proliferation assays. Molecule release (cytokines or cytolytic granule contents) from activated CD8^+^ or CD4^+^ T cells can be evaluated by different methods. Intracellular staining of induced molecules detects specific T-cells on a single cell level whereas CBA measures secreted proteins produced by bulk cell populations. ELISPOT detects secretion “spots” of a specific mediator and quantifies the magnitude of this response. Monoclonal antibodies for phenotyping can be used to characterize specific T cells.

Despite the translational challenges of animal models in Chagas disease (24, 25), they are still crucial for increasing the knowledge of the disease and the interaction between the host and the parasite (26–29). Indeed, mouse models (wild type mice with H2 –mouse MHC- molecules or transgenic ones expressing human MHC) are broadly utilized under different strategies to evaluate the specificity of T cell response against *T. cruzi* infection (**Figure 3**). Importantly, human samples, especially blood, are often used to validate results obtained from animal models and to analyze the particularities of human T cell responses against the parasite (**Figure 3**). In this regard, the HLA (Human Leukocyte Antigen, human MHC molecules) restriction ultimately determines the repertoire of peptides that are presented for interaction with the TCR, limiting the usefulness of non-human models to evaluate T cell specificity at the epitope level.

## CD8^+^ T Cell Specificity in Chagas Disease

Several T CD8^+^ epitopes have been described and confirmed as immunogens in Chagas disease patients, being those derived from the *trans*-sialidase proteins family widely assessed (30–33). Most studies focus on MHC class I presentation restricted to HLA-A*02 alleles because of its high prevalence in individuals of various ethnic backgrounds, including those living in areas where Chagas disease is endemic (34, 35).

Within the TS family, immunogenic CD8^+^ T cell epitopes identified in HLA-A*02:01^+^ patients belong predominantly to trypomastigote surface antigen (TSA)-1, amastigote surface proteins (ASP)-1 and -2 and flagellum-associated protein FL-160 (**Table 1**). The first studies on the role of these antigens in Chagas disease were performed in mouse models and showed protective responses against *T. cruzi* infection after vaccination with antigen-encoding plasmids (53–55). Additionally, CD8^+^ cytotoxic T cells epitopes from those proteins (**Table 2**), restricted to the mouse H-2K^b^ class I molecule, sensitized RMA-S target cells (a TAP deficient cell line derived from the Rauscher leukemia virus-induced mouse T-cell lymphoma RBL-5) for lysis by specific spleen cells from *T. cruzi*-infected mice (55–57). The antigen-specific CD8^+^ cytotoxic T cells response was also demonstrated in *T. cruzi*-infected or peptide-immunized A2.1/K^b^ transgenic mice (expressing a chimeric class I molecule consisting of the human HLA-A2.1 α1 and α2 domains ligated to the mouse α3, transmembrane and cytoplasmic H2-K^b^ domains). Splenocytes from these animals were submitted to cytotoxicity assays, in which Jurkat A2.1/K^b^ or lymphoblastoid 721.221 A2.1/K^b^ targets cells were sensitized with TSA-1-, ASP-1- and ASP-2-derived peptides (36). Furthermore, specific CD8^+^ T cell response against peptides from these antigens has been also observed in peripheral blood mononuclear cells (PBMC) from HLA-A*02:01^+^ donors with chronic *T. cruzi* infection (32, 36) (**Table 1**). Interestingly, the cytotoxic activity induced by selected epitopes from ASP-1, ASP-2 and TSA-1 was reduced by the depletion of CD8^+^ T cells, but not CD4^+^ T cells, in samples from individuals living in endemic regions in Guatemala (36). Additionally, assays using MHC tetramers bound to some of those peptides demonstrated a low frequency of peptide-specific CD8^+^ T cells in HLA-A*02:01^+^ *T. cruzi*-infected patients residing in non-endemic areas of Argentina at the time of sample collection (32).

**Table 1:** *T. cruzi* epitopes recognized by T cells from Chagas disease patients.

^a^Epitope	^b^ID	^c^Antigen Name	T cell/HLA Restriction	^d^Recognition by T cells in Mouse Models	Reference
VLAKDGTEV IAGGVMAVV FVNHDFTVV LLLLGLWGL LLGLWGLTGL	ASP-1_50_ ASP-1_71_ ASP-1_508_ ASP-1_666_ ASP-1_668_	Surface protein 1/*trans*-sialidase, putative/amastigote surface protein-1 (ASP-1)	CD8^+^/ HLA-A*02:01	Yes (A2.1/K^b^ Tg mice)	(32, 36)
WVFPESISPV FVNHRFTLV	ASP-2_302_ ASP-2_551_ (TSA2-37)	Surface protein 2/*trans*-sialidase, putative, Group II/amastigote surface protien-1 (ASP-2)	CD8^+^/ HLA-A*02:01	Yes (A2.1/K^b^ Tg mice)	(30, 32, 33, 36)
KLFPEVIDL FVDYNFTIV VLLPSLFLL VLLPSLFLLL	TSA-1_89_ TSA-1_514_ TSA-1_818-826_ TSA-1_818-827_	Trypomastigote surface glycoprotein/*trans*-sialidase, putative/trypomastigote surface antigen-1 (TSA-1)	CD8^+^/ HLA-A*02:01	Yes (A2.1/K^b^ Tg mice; HHD Tg mice)	(32, 36, 37)
LLGLWVFAAL GLLPSLLLLL RLLPSLLLLL LLGLWGTAAL FLSHDFTLV SLSHYFTLV FLSHNFTLV FVNYDFALV FANCNFTLV FANHDFTLV FVSCDFTIV FANHKFTLV FANNKFTLV FANYKFTLV FVNYNFTLV FANHNFTLV FVDYNFSLV FANYNFTLV IANYNFTLV FANNEFTLV FVNYDFTIV ELLRPTTLV DVSRPTAVV	TSA2-2 TSA2-3 TSA2-3.2 TSA2-13 TSA2-24 TSA2-26 TSA2-27 TSA2-28 TSA2-32 TSA2-34 TSA2-35 TSA2-38 TSA2-43 TSA2-44 TSA2-45 TSA2-46 TSA2-47 TSA2-48 TSA2-48.3 TSA2-49 TSA2-50 TSA2-52 TSA2-66	*Trans*-sialidase, putative	CD8^+^/ HLA-A*02:01	Yes (A2.1/K^b^ Tg mice)	(30, 33)
VTDNNRSFY LWLTDNTHI LLLGLWGFA YNFTLVATV FTSAVLLLL MLVTLPVYS NVMLVTLPV FVSPSLVSA ALSSSLGNV HLFYSAVLL FLYNRPLNS HNFTLVASV LLLLVVMMCC TSAVLLLLVV SIPTAGLVAV FQGAWAEWPV RVLLLLLLGL MLSRVAAVK FTLVATVSI FTLVASVTI AVAEAQCKK VALMLQGNK LVTLPVYSK DVAASSLLY ITATIEGRK IYMLVGKYS GVIAAFAEGH		*Trans-*sialidase, putative	CD8^+^/ HLA-A1 HLA-A2 HLA-A2 HLA-A2 HLA-A2 HLA-A2 HLA-A2 HLA-A2 HLA-A2 HLA-A2 HLA-A2 HLA-A2 HLA-A2 HLA-A2 HLA-A2 HLA-A2 HLA-A2 HLA-A3 HLA-A3 HLA-A3 HLA-A3 HLA-A3 HLA-A3 HLA-A3 HLA-A3 HLA-A3 HLA-A3	Not tested	(30)
FTSAVLLLV VMMSCSSEA SMNATLVQA RLLSPTTIV KVVSLVILA AASTLLYATV TLLYATVEV KLYCSYEVA WLSDCGEAL KTWADEYLCV FTLVAPVSI IILNGSLLTL QMDYSNGLFV GLLLLGLWGT LLLGLWGTA	FL _6–14_ FL _16–24_ FL _47–55_ FL _120–128_ FL _236–244_ FL _377–386_ FL _380–388_ FL _401–409_ FL_457– 465_ FL _480–489_ FL _534–542_ FL _581–590_ FL _610–619_ FL _990–999_ FL _992–1000_	Flagellum-associated protein/*trans*-sialidase, putative, Group III/FL-160-1	CD8^+^/ HLA-A*02:01	Not tested	(31)
ALSLAAVLV ALSLAAVLVV VVMACLVPAA VMACLVPAA SVFRENLFL GLMNNAFEWI LMNNAFEWI LMNNAFEWIV WIIKNSWTA VTLPTGQCL LLTTSGVSA	CZ _7–15_ CZ _7–16_ CZ _15–24_ CZ_16–24_ CZ_60–68_ CZ _188–197_ CZ _189–197_ CZ _189–198_ CZ _300–308_ CZ _378–386_ CZ _386–394_	Cysteine peptidase, putative, cathepsin L-like protein, putative	CD8^+^/ HLA-A*02:01	Not tested	(31)
RQRRYQPYHSRHRRL		Cruzipain/cysteine peptidase, putative/major cysteine protease	CD8^+^/ HLA-A*31:01	Not tested	(38)
AVPEVTDVTL KLEKIEDEL RLYKTLGQL	PFR2_19–28_ PFR2_156–163_ PFR2_449–457_	Major paraflagellar rod protein/paraflagellar rod protein 2 (PFR2)	CD8^+^/ HLA-A*02:01	Yes (A2.1/K^b^ Tg mice)	(39, 40)
FVSCCGELTV DIIEQMKGV GVSGVINAL	PFR3_428–436_ PFR3_475–482_ PFR3_481–489_	Paraflagellar rod component, putative	CD8^+^/ HLA-A*02:01	Yes (A2.1/K^b^ Tg mice)	(39, 40)
TLEEFSAKL	KMP-11_4−12_ (KP-1 or TcTLE)	Kinetoplastid membrane protein-11 (KMP-11)	CD8^+^/ HLA-A1 (A*0101, A*3201) HLA-A2 (A*0201, A*0205, A*0222, A*0226, A*0259, A*0287, A*6802) HLA-A24 (A*2402, A*2301)	Yes (A2.1/K^b^ Tg mice)	(40–46)
TLLTIDGGI TLQPVERVL	HSP70_210-8_ HSP70_316-24_	Heat shock protein-70 (HSP-70)	CD8^+^/ HLA-A*02:01	Yes (A2.1/K^b^ Tg mice)	(47)
AAAGDKLSL TVFDASRSTV ALRNLRVFL ALQVTNHRYL	TcCA-2_273-281_ TcCA-2_442-451_ TcCA-2_607-615_ TcCA-2_657-666_	Surface antigen 2 (CA-2)/TcCA-2	CD8^+^/ HLA-A*02:01	Not tested	(40, 48)
KPPPFGQAAAGDKPS KPSPFGQAAAGDKPP KPSPFGQAAAGGKPP KPPPFGQAAAGDKPP KPPPFGQAAEGDKPP KPPPFGQAAAADKPS KPSLFGQAAAGDKLS KLSLFGQAAAGDKPP KPPPFGQAAAGDKPA KPAPFGQAAEGDKPP	S15.1 S15.2 S15.3 S15.4 S15.5 S15.6 S15.7 S15.8 S15.9 S15.10	B13 antigen Putative, surface antigen-2 (TcCA-2)	CD4^+^/HLA-DQA1*0501/ DQB1*0301 (HLA-DQ7)	Not tested	(49–51)
SADNTNSGAGGGLSSKAFEWIVQENNGAVYTED		Cruzipain, cysteine peptidase	CD4^+^/ HLA-class II	Not tested	(52)
VECQWFLAGHPLTNLS	TcCZp1	Cruzipain, cysteine peptidase, putative, cysteine peptidase, clan CA, family C1, cathepsin L-like, putative	CD4^+^/ HLA-class II	Not tested	(38)
ENQLYHFANYKFTLV	TcTSp1	Putative trans-sialidase, Group V	CD4^+^/ HLA-class II	Yes (A2.1/K^b^ Tg mice)	(38)
TVPYHFANSKFTLVA	TcTSp2	Putative trans-sialidase, Group III/Flagellum-associated protein FL-160-2	CD4^+^/ HLA-class II	Not tested	(38)
MLSLVAAVKAPRTHN	TcTSp3	Putative trans-sialidase, Group V	CD4^+^/ HLA-class II	Not tested	(38)
GVVMEDGTLVFPLMA	TcTSp4	Putative trans-sialidase, Group II	CD4^+^/ HLA-class II	Not tested	(38)
HRFTLVATVTIHQVPK	TcTSp5	ASP-2/*trans*-sialidase, putative, Group II	CD4^+^/ HLA-class II	Not tested	(38)

a Underlined sequences are evaluated in more than one publication referenced.

b ID from publication.

c Antigen name from NCBI database and the publication referenced.

d Epitope recognition by T cells from a mouse model besides T cells from patients. Not tested refers to those peptides which are not evaluated in mouse models in publications referenced. HHD transgenic (Tg) mice express a chimeric HLA-A2 molecule consisting of human β_2_-microglubulin and human HLA-A2.1 α1 and α2 domains fused to the α3, transmembrane and cytoplasmatic domains of the mouse H2-D.

**Table 2:** *T. cruzi* epitopes recognized by CD8^+^ T cell from mice (not tested in Chagas disease patients).

^a^Epitope	^b^ID	^c^Antigen Name	^d^Mouse Model	EMHC Restriction	Reference
VDYNFTIV	Pep 77.2^#^ (TSA-1_515-522_)	Trans-sialidase, putative/ surface glycoprotein/ trypomastigote surface antigen-1 (TSA-1)	CL57BL/6	H-2K^b^	(55, 56)
VNHDFTVV	PA14^#^	Putative trans-sialidase, Group II/surface protien-1/amastigote surface protein-1 (ASP-1)	CL57BL/6	H-2K^b^	(57)
KNYPFSSI NTLVFPLV DNRQYSFV VNHRFTLV EKEANALYLWV	PA5 PA6 PA7 PA8^#^ PA10	Putative trans-sialidase, Group II/surface protein-2/amastigote surface protein-2 (ASP-2)	CL57BL/6	H-2K^b^ H-2K^b^ H-2K^b^ H-2K^b^ H-2K^b^/H-2D^b^	(57)
LSHNFTLV ANYDFTLV ANYKFTLV ANYNFTLV LSHDFTVV LSHNLTLV LSHSFTLV VNYDFTLV ANYNFTLL LLRPTTLV VNYDFTIV VGRPTTVV LHKRFTLV AKYNFTLL ANYKFTLL ANYNLTLV ANHRFTLV	TSKB4^#^ TSKB18 TSKB20^#^ TSKB21^#^ TSKB24 TSKB27 TSKB60 TSKB74 TSKB80 TSKB84 TSKB89^#^ TSKB92 TSKB96 TSKB252 TSKB260 TSKB306 TSKB388	*Trans*-sialidase, putative	CL57BL/6 TSKB20, TSKB18, TSK20/18 Tg mice (58)	H-2K^b^	(33, 58–63)
EVYSLVFARL NVLLYNRPL WLTDNTHIV NTWRDEYLGV KVGSDVFAV RVFTSAVLL	F-TS-2 NF-TS-2 NF-TS-3 NF-TS-4 NF-TS-5 NF-TS-6	*Trans*-sialidase, putative	HHD Tg mice	HLA-A*02:01	(64)
FADKPDESTLS HTDKFNKKM	KMP-11_41-50_ (K3) KMP-11_58-66_ (K4)	Kinetoplastid membrane protein-11 (KMP-11)	A2.1/K^b^ Tg mice	HLA-A*02:01	(41, 42)
YEIQYVDL RVVSFTQM	PFR-1_164−171_ PFR-3_123–130_	Paraflagellar rod component/paraflagellar rod protein	CL57BL/6 Cd8a^tm1Mak^	H-2K^b^	(65)
GVPVDPSRV YLAAYALVGL	P1 P7	Ribosomal P2 type protein/TcP2β	HHD Tg mice	HLA-A*02:01	(37)
SLFGYRKL	Tcβ3 P1	Adaptin AP-3 complex β3 subunit Tcβ3	CL57BL/6	H-2K^b^	(66)
PAALFKEL	FCaBP p4	Flagellar Ca^+2^ binding protein	CL57BL/6	H-2K^b^	(66)
ELTMYKQLL	LYT1 p5	LYT-1	CL57BL/6	H-2K^b^	(66)
SVPIRLLVL LGFQERNVL	GFTKB-16 GFTKB-17	β-galactofuranosyl transferase	CL57BL/6	H-2K^b^	(33)
PSVRSSVPL VPLNKCNRL	CRZPKB-5 CRZPKB-9	Cruzipain/cysteine peptidase	CL57BL/6	H-2K^b^	(33)
DSLTNLRAL RIPKVMQLV	HSP7021_255-263_ HSP7021_345-53_	Heat shock protein-70	A2/K^b^ Tg mice	HLA-A*02:01	(47)
VVMACLVPA	Non-TS 2	Cruzipain precursor/cruzipain/cysteine peptidase	HHD Tg mice	HLA-A*02:01	(64)
TLLFQVLLL RIGDVCAEV LLFQVLLLC AVFDSVYSV TLMDFCPYI	Non-TS 3 Non-TS 4 Non-TS 5 Non-TS 25 Non-TS 33	Surface protease GP63	HHD Tg mice	HLA-A*02:01	(64)
YVVSLLADT SVVSVFFLL	Non-TS 6 Non-TS 29	Histone H3	HHD Tg mice	HLA-A*02:01	(64)
ALHSLVLFL SLFPFFFFV ALLPWLLVL FLIFRFMVL FMACHTNPI FLNQFGTRL	Non-TS 7 Non-TS 9 Non-TS 13 Non-TS 17 Non-TS 18 Non-TS 26	Hypothetical protein	HHD Tg mice	HLA-A*02:01	(64)
AVIPSTFPL	Non-TS 8	ATP synthase	HHD Tg mice	HLA-A*02:01	(64)
FLVDTIYSI	Non-TS 10	Citrate synthase	HHD Tg mice	HLA-A*02:01	(64)
YLDAVFYPL	Non-TS 11	Pitrilysin-like metalloprotease	HHD Tg mice	HLA-A*02:01	(64)
SLLCVISFI SMNAGLYLL	Non-TS 12 Non-TS 24	Retrotransposon hot spot protein	HHD Tg mice	HLA-A*02:01	(64)
LMNDVWFSL	Non-TS 16	Heat shock protein 70	HHD Tg mice	HLA-A*02:01	(64)
MMHPFLCAL	Non-TS 19	Surface protein ToIT	HHD Tg mice	HLA-A*02:01	(64)
SMQEYRHMV	Non-TS 27	Dynein light chain	HHD Tg mice	HLA-A*02:01	(64)
QMLHNVASL	Non-TS 31	Pumilio/PUF RNA binding protein	HHD Tg mice	HLA-A*02:01	(64)
MMMTGRVLL	Non-TS 32	Mucin-associated surface protein	HHD Tg mice	HLA-A*02:01	(64)

a Underlined sequences are evaluated in more than one publication referenced.

b ID from publication. (#) symbol refers to epitopes contained in epitopes recognized by T cells from Chagas disease patients.

c Antigen name from NCBI database and the publication referenced.

d Mouse models used for the analysis of T cell specificity. Cd8a^tm1Mak^ mice lack surface expression of CD8 due to the disruption of Lyt-2 (Cd8a), the gene coding for the T-cell surface glycoprotein CD8 α chain. TSKB20, TSK18 and TSK20/18 Tg mice express TSK20, TSK18 or both T. cruzi epitopes from TS proteins as self antigens.

e MHC restriction is indicated in wild type (H2-K or H2-D) and Tg (HLA-A*02:01) mice. In assays with Tg mice, animals are immunized with the antigen and their splenocytes stimulated with APCs expressing HLA-A2.1 molecules pulsed with the same antigen.

Studies based on peptides predicted to bind H-2K^b^ MHC molecules showed a CD8^+^ T cell response that is dominated by epitopes encoded by *trans*-sialidase family genes (33) (**Table 2**). Frequencies of IFN-γ-producing cells were lower against non-TS epitopes, and target splenocytes from naïve mice loaded with non-TS epitopes were poorly recognized in *in vivo* cytotoxic T lymphocyte assays (33). Similarly, the immunization of C57BL/6 mice with pools of *trans*-sialidase but not mucin genes provided protection against an otherwise lethal *T. cruzi* challenge (66). Moreover, the CD8^+^ T cell response focused on a few epitopes (33, 59–61) was interpreted in favor of the hypothesis of immunodominance against certain peptides as mechanism to evade the immune response (62). Nonetheless, experimental approaches with mouse models later demonstrated that the immune control of *T. cruzi* infection was independent of the recognition of the therein called “dominant” TSKB20 and “subdominant” TSKB18 epitopes (58, 63). In fact, despite the widespread idea of TS broad dominance of the T cell response against *T. cruzi*, there is insufficient evidence of a correlate in the context of human infection.

A similar approach to that described for the prediction and selection of H-2K^b^–binding peptides allowed the determination of TS peptides (**Table 1**) that induced diverse levels of reactivity in IFN-γ ELISPOT experiments for HLA-A*02:01^+^ chronic Chagas disease patients (33). Interestingly, many TS peptides (**Table 1**), which were found by *in silico* prediction of their binding affinity to MHC from different HLA allele supertypes (HLA-A1, -A2, -A3, -A24, -B7 and -B44), showed a positive outcome in IFN-γ ELISPOT assays, being A2 supertype peptides the most frequently recognized (30).

Additional CD8^+^ T cell targets were identified from enzymatically active TS (F-TS), non-functional TS (NF-TS) and non-TS proteins (**Table 2**) after vaccination of HHD transgenic mice with dendritic cells pulsed with peptide pools (64). Furthermore, HLA-A*02:01-restricted epitopes from cruzipain (also known as GP 57/51 or cruzain, a cysteine peptidase) and the FL-160 protein (renamed FL-160-1, the 160 kDa flagellum-associated surface protein of trypomastigotes) were described (31). Peptides from those proteins (**Table 1**) were recognized by PBMC from chronic Chagas disease patients in IFN-γ ELISPOT and tetramer assays (31). Another cruzipain epitope was described in a recent publication of our group using an *in silico* guided approach (38). Sequences of *T. cruzi* proteins characterized as T cell immunogens in the Immune Epitope Database [IEDB-, www.iedb.org (67)] were screened and the binding to a set of HLA-A and HLA-B or HLA-DRB1 molecules were predicted. One out of the four novel peptides reported (namely TcCZp2, **Table 1**), revealed predominant CD8^+^ T cell activation in IFN-γ ELISPOT assays with PBMC depleted of CD4^+^ T cells (38). In addition, the predicted nested core of this peptide (TcCZp2me) allowed the identification of epitope-specific CD8^+^ T cells in HLA-A*31:01^+^ patients, with a predominant effector memory T_EMRA_ (terminal effector T cells re-expressing CD45RA) or T_EM_ (effector memory) cell phenotype (38).

Potential CD8^+^ T cells targets were also described in the paraflagellar rod proteins (known as PFR or TcPRP), located at the *T. cruzi* flagellum and specific to the kinetoplastids. Mice receiving injections of these proteins (namely PAR1 or PFR-3 and PAR2) had an immune response capable of reducing the level of circulating parasites, and improved survival (68). Another research on the subject showed that cytotoxic T lymphocyte lines generated from chronically *T. cruzi*-infected mice specific for peptides PFR-1_164−171_ and PFR-3_123–130_ (**Table 2**) had high levels of lytic activity and secreted IFN-γ in response to parasite-infected target cells. Moreover, CD8 knockout (Cd8a^tm1Mak^) mice deficient in functional cytotoxic T cells, immunized with PFR and challenged with *T. cruzi*, showed higher parasitemia and lower survival than PFR-immunized control mice (65). DNA immunization with the PFR2/heat shock protein 70 (HSP70) fused genes not only triggered the cytotoxic activity of antigen-specific CD8^+^ T cells but also provided a protective response against a *T. cruzi* infection (69). Furthermore, the immunization of C57BL/6-A2/K^b^ transgenic mice with plasmids encoding the fusion proteins PFR2-HSP70 and PFR3-HSP70 induced a specific CD8^+^ cytotoxic T cells response against epitopes PFR2_449–457_ and PFR3_481–489_ (39). Regarding the response in the human host, peptides containing potential HLA-A*02:01-binding sites were identified by the analysis of *T. cruzi* PFR2 and PFR3 using the algorithms SYFPEITHI [www.syfpeithi.de, (70)] and BIMAS [www-bimas.cit.nih.gov, (71)] (39). Peptides from each protein (**Table 1**) were recognized by PBMC from chronic Chagas disease patients and peptide-specific CD8^+^ T cells exhibited a pro-inflammatory cytokine secretion profile (IFN-γ, TNF-α and IL-6). Remarkably, cytotoxic activity was observed in CD8^+^ T cells from asymptomatic patients but not in chronic patients with cardiomyopathy (39). Interestingly, some epitopes from PFR and other paraflagellar rod component proteins were described in a recent *in silico* study based on the identification of conserved epitopes (72).

Another vastly described *T. cruzi* protein containing CD8^+^ T cell epitopes is the kinetoplastid membrane protein-11 (KMP-11), which has been reported to be mainly located in the flagellar pocket. The first investigations into CD8^+^ T cell response against this antigen were carried out in mouse models. Immunization of A2.1/K^b^ transgenic mice with fused *T. cruzi* KMP-11/HSP70 genes generated a population of reactive CD8^+^ cytotoxic T cells against KMP-11 peptides (named K1, K3 and K4) (**Table 2**) containing predicted HLA-A*02:01 binding motifs (41, 42). With regard to humans, HLA-A*02:01^+^ chronic Chagas disease patients from endemic zones exerted a CD8^+^ T cell specific response towards the TcTLE peptide [previously known as K1 peptide, **Table 1**, (43)]. This peptide was also recognized by infected patients carrying HLA-A2 supertype alleles other than HLA-A*02:01 (A*0205, A*0222, A*0226, A*0259 and A*0287). Both HLA-A*02:01^+^ and HLA-A*02:01^-^ infected patients had TcTLE-specific CD8^+^ T cells displaying a phenotype compatible with differentiated T_EM_ cells (44). Moreover, the introduction of specific mutations in TcTLE showed limited effect on the functional activity of CD8^+^ T cells from HLA-A*02:01^+^ chronic Chagas disease patients (45). TcTLE also exhibited a promiscuous recognition in patients with Chagas disease expressing different HLA-A supertypes (HLA-A1, -A2 and -A24). Remarkably, the evaluation of IFN-γ, TNF-α, IL-2, perforin, GrB and CD107a/b after stimulation with this peptide demonstrated that HLA-A2^-^ patients had mono- and polyfunctional (simultaneously expressing multiple cytokines and exhibiting cytotoxic activity at the single-cell level) CD8^+^ T cells and their frequency could be associated with disease severity (46). Similar results had been previously described when PBMC from patients at different stages of chronic Chagas disease were stimulated with KMP-11 recombinant protein. In fact, patients with less severe disease showed a higher frequency of polyfunctional KMP11-specific CD8^+^ T cells whereas patients at advanced stages of the disease demonstrated a higher frequency of monofunctional CD8^+^ T cells (73).

Class-I epitopes recognized by patient cells were identified in other *T. cruzi* proteins, such as HSP70 and the putative surface antigen TcCA-2. Two *T. cruzi* HSP70 CD8^+^ epitopes were shown to be processed and presented during natural infection. Peptides predicted with the algorithms SYFPEITHI and RANKPEP [http://immunax.dfci.harvard.edu/Tools/rankpep.html (74),] were recognized by cells from A2.1/K^b^ transgenic animals immunized with the HSP70 recombinant protein or infected with the parasite. Two of those peptides (HSP70_210-8_ and HSP70_316-24_,**Table 1**) recognized by CD8^+^ T cells of HLA-A*02:01^+^ Chagas disease patients from endemic areas residing in a non-endemic zone induced peptide-specific cytotoxic activity and pro-inflammatory cytokine (IFN-γ and TNF-α) secretion (47). With regard to the *T. cruzi* carbonic anhydrase protein TcCA-2, four CD8^+^ epitopes (TcCA-2_273-281_, TcCA-2_442-451_, TcCA-2_607-615_ and TcCA-2_657-666_) (**Table 1**) were found to be processed and presented during Chagas disease in a multiplexed cytokine secretion assay (48). CD8^+^ T cell specific for these epitopes were functionally active in both asymptomatic and symptomatic cardiomyopathy chronic Chagas disease patients (from endemic areas but residing in a non-endemic zone) (48). In addition, TcCA-2_442-451_ and TcCA-2_607-615_ peptides allowed the characterization of different phenotypical profiles in cells from asymptomatic *versus* cardiac Chagas disease patients. Thus, the TcCA-2-specific CD8^+^ T cells from patients with cardiac symptoms were mainly effector memory cells (T_EM_ or T_EMRA_) whereas those present in the asymptomatic phase were predominantly naïve T cells (48). In a recent study, the same two peptides from TcCA-2, and those from PFR2 (PFR2_449-457_), PFR3 (PFR3_428-436_) and KMP-11 (K1) (**Table 1**), were used to evaluate the antigen-specific CD8^+^ T cell response in asymptomatic chronic Chagas disease patients, both before and after benznidazole treatment (40). Although different outcomes for memory phenotype, senescence and antigen experience were found depending on the peptide analyzed, CD8^+^ T cells specific to all the peptides showed a high percentage of these cells at an advanced stage of differentiation (CD45RA^+^CD127^-^) both before and after treatment (40). In a prior report, the same group had characterized the functional capacity of antigen-specific CD8^+^ T cells (based on the expression of IFN-γ, IL-2, TNF-α, granzyme B and perforin) before and after drug treatment but, in this case, they used recombinant proteins instead of peptides from those *T. cruzi* antigens (75). Patients showing therapeutic efficacy (based on serological response) presented polyfunctional antigen-specific CD8^+^ T cells whereas cells from patients with therapeutic failure were monofunctional (75).

Other targets for the CD8^+^ T-cell response were described in mouse models, but without confirmed immunogenicity in humans (**Table 2**). The sequences for flagellar Ca^2+^ binding protein (FCaBP), LYT-1 and AP-3 were scanned for peptides that matched the mouse H-2K^b^ allele-specific class I peptide binding motif. LYT-1, a protein predicted to be secreted by trypomastigotes, was found to be a target of cytotoxic T cell responses in chronically infected mice, and the immunization with a plasmid encoding a peptide from this protein protected mice from lethal *T. cruzi* challenge (66). Peptides from FCaBP and a homologue of the adaptin AP-3 complex β3 subunit (Tcβ3) were recognized by spleen cells from infected mice, and these lysed peptide-pulsed RMA-S target cells (66). With regard to ribosomal P2β protein (TcP2β), two peptides (TcP2-P7 and TcP2-P1, **Table 2**), containing known binding motifs for MHC molecule encoded by the HLA-A*02:01, elicited lytic activity when HHD transgenic mice were immunized with them individually (37).

## CD4^+^ T Cell Specificity in Chagas Disease

Only a few proteins have been demonstrated to induce a recall response on CD4^+^ T cells from *T. cruzi* infected-individuals in the chronic phase of the disease (38, 49, 52, 76, 77).

Cruzipain elicited proliferative responses of PBMC from chronic Chagas disease patients both in its recombinant and isolated (from *T. cruzi* epimastigotes) forms. Moreover, T cell lines generated from PBMC using those antigens consisted of CD4^+^ T cells, and their stimulation with recombinant or isolated cruzipain induced their production of IFN-γ. Furthermore, anti-cruzipain T cell lines showed a strong proliferative response against one out 11 peptides spanning portions of the catalytic domain of the cruzipain protein [**Table 1** (52)].

The recognition of the tandemly repetitive *T. cruzi* B13 protein (a homolog of TcCA-2) was also characterized by T cell lines, clones and PBMC from chronic Chagas disease patients (49). The PBMC proliferative response to recombinant B13 protein was similar between cardiac and asymptomatic patients and among non-infected control individuals. Likewise, when the proliferative response to B13-derived peptides (**Table 1**) was analyzed in HLA-DQ7^+^ subjects, peptide S15.4 was mainly recognized by patients with cardiomyopathy whereas S15.1 and S15.3 peptides more frequently targeted by cells from asymptomatic donors, and peptide S15.9 triggered a response in cells from both groups of Chagas disease patients (49). Additionally, a CD4^+^ T cell clone from an HLA-DQ7^+^ individual responder to the peptide S15.4 presented cross-reactivity against peptides from human β-cardiac myosin heavy chain according to the central HLA-DQ7 binding motif (50). Interestingly, proliferation assays on PBMC from S15.7-responder HLA-DQ7^+^ individuals revealed that peptide S15.7 was recognized by PBMC from all subjects tested whereas none bound to its highly resistant to proteolytic digestion analogues (51).

Concerning KMP-11, a specific CD4^+^ T cell response was shown as INF-γ production upon the stimulation of PBMC from chronic Chagas disease patients with the recombinant antigen (76). KMP-11 responders were mostly patients with severe cardiomyopathy (abnormal ECG and cardiomegaly in chest radiography) whereas only one subject was determined as responder out of eight evaluated donors in patients with normal findings on ECG, or abnormal ECG and no cardiac enlargement (76).

On the other hand, TSA-1 and Tc24 (flagellar calcium binding protein of 24 kDa) were evaluated for the ability to recall CD4^+^ T cell response in chronic Chagas disease patients (77). Results demonstrated a higher proportion of CD4^+^ T cells that proliferated after stimulation with rTc24, whereas CD3^+^ T cells responded when rTSA-1 was used as stimulus (77).

A recent in *silico* approach also enabled the identification of potential *T. cruzi*-specific CD4^+^ epitopes from a co-chaperone GrpE and from a hypothetical protein (72). And more recently, another predictive approach developed by our group led to the discovery of peptides demonstrated to induce CD4^+^ T cell response in Chagas disease patients (38). In fact, these cells were the leading producers of IFN-γ in response to the novel *T. cruzi* peptides TcTSp3, TcTSp4 and TcTSp5 (**Table 1**). Notably, the IFN-γ responses against TcTSp1, TcTSp2 and TcCZp1 (**Table 1**) were also predominantly attributed to CD4^+^ T cell activation, although they comprised sequences from cruzipain and TS proteins previously reported to induce CD8^+^ T cell response in Chagas disease or to bind HLA-A*02:01 (31, 33). Regarding phenotype characterization, CD4^+^ T cells showed heterogeneous degree of maturation between chronic Chagas´ disease patient population, although central memory (T_CM_) T cells seemed to be preponderant in patients with IFN-γ response against TcTSp4 (38).

## Concluding Remarks and Future Perspectives

Experimental animal models and studies with clinical samples have shown that CD4^+^ and CD8^+^ T cells play a crucial role in the control of Chagas disease. For this reason, elucidating their specificity in relation with their functional features represents an opportunity for a deeper understanding of the immune response against *T. cruzi* infection. Up to now, almost 200 T cell epitopes from 49 *T. cruzi* antigens have been described (IEDB, accessed February 2021) whereas the haploid *T. cruzi* genome has more than 10,000 protein coding genes (78). Thus, continued efforts are required to explore the role of the majority of *T. cruzi* proteome in the host immune response.

Taking into account the disadvantages of treatments with current drugs (benznidazole and nifurtimox), which have an unclear efficacy at chronic stage (79–81), the identification of *T. cruzi* antigens could contribute not only to discover putative biomarkers for the follow-up of Chagas disease patients, but also to develop vaccines based on the induction of antigen-specific T cells. These could be an attractive and cost effective alternative (or complement) to current drug-based therapies. Therefore, it is crucial to consider the high biological complexity of *T. cruzi* and the huge diversity of HLA haplotypes, the major factors determining the repertoire of an individual´s T cell specificities. Targeting as many antigens as possible in a vaccine design could provide a way to overcome the limitations imposed by host diversity.

In this regard, *in silico* analyses to identify proteins conserved across diverse *T. cruzi* strains and expressed in stages of the parasite life cycle relevant to human infection, constitute useful approaches to narrow down the universe of *T. cruzi* antigens to a set of candidates addressable by laboratory scale immunoassays. However, these methods come with caveats and pitfalls of their own. Up to date, reports about antigen-specific T cell response against *T. cruzi* infection have focused on predicting peptide binding to MHC. Despite this prediction is very useful to limit the number of potential T cell epitopes to test experimentally, not all MHC presented peptides are immunogenic. Consequently, the TCR:pMHC interaction should be better understood in order predict MHC restricted peptides that actually become T cell epitopes. Remarkably, this issue may result challenging due to the promiscuity of TCRs (82), the limited information about the patterns that govern T-cell recognition and the ability of TCRs to cross-recognize structurally related elements (83). In general, extensive data on peptides-MHC binding is available, whereas there is less information linking specific TCRs to their cognate target (84). In this context, different approaches have been proposed to gain knowledge on TCR:pMHC interaction (84–89). Nonetheless, none of them has so far been used to analyze specific T cell response in Chagas disease.

In parallel with immunobioinformatics methods which have powered the search for epitope-based vaccines candidates against *T. cruzi* (72, 90), other promising and newer tools may be leveraged to detect MHC-bound peptides relevant to the parasite-specific T cell response. These include pipelines used for neoantigen discovery (91) and immunopeptidomics approaches (92–94), which consist in the isolation of peptides from the pMHC complexes purified from the APC surface and their identification by mass spectrometry and bioinformatics.

Taken together, our review highlights the importance of the specific T cell response in Chagas disease. Relevant information about peptides involved in this response has been accumulating across decades of research. Nonetheless, the available data is so far insufficient to effectively enable the translation of this knowledge into treatment strategies for this neglected disease. In this regard, attempts to successfully reach optimal solutions for the millions of people affected are still needed and emerging techniques employed in the study of other diseases are highly promising to this end.

## Author Contributions

FF, GA, and KG contributed to conception of the revision. FF organized the data and wrote the first draft of the manuscript. All authors contributed to the article and approved the submitted version.

## Funding

This work was supported by Consejo Nacional de Investigaciones Científicas y Técnicas (CONICET; PIP Number 112-2015010-0547).

## Conflict of Interest

The authors declare that the research was conducted in the absence of any commercial or financial relationships that could be construed as a potential conflict of interest.

## Abbreviations

ASP, amastigote surface protein; APC, antigen presenting cell; CZ, cruzipain; ELISPOT, enzyme-linked immunospot; FCaBP, flagellar Ca^2+^ binding protein; FL-160, 160 kDa flagellum-associated surface protein; HLA, human leukocyte antigen; HSP70, heat shock protein 70; IEDB, Immune Epitope Database; KMP-11, kinetoplastid membrane protein-11; MHC, major histocompatibility complex; PBMC, peripheral blood mononuclear cell; PFR or PAR, paraflagellar rod protein; pMHC, peptide-MHC complex; TCR, T cell receptor; Tc24, flagellar calcium binding protein of 24 kDa; TcCA-2, surface antigen-2; T_EM_, effector memory T cell; T_EMRA_, terminal effector T cells re-expressing CD45RA; T_H_, helper T cell; T_reg_, regulatory T cell; T_scm_, stem cell memory T cell; TS, *trans*-sialidase; TSA, trypomastigote surface antigen.
